# The association of low serum albumin level with severe COVID-19: a systematic review and meta-analysis

**DOI:** 10.1186/s13054-020-02995-3

**Published:** 2020-05-26

**Authors:** Muhammad Aziz, Rawish Fatima, Wade Lee-Smith, Ragheb Assaly

**Affiliations:** 1 Department of Internal Medicine, University of Toledo Medical Center, Toledo, OH USA; 2 University of Toledo Libraries, University of Toledo Medical Center, Toledo, OH USA; 3 Division of Pulmonary and Critical Care Medicine, University of Toledo Medical Center, Toledo, OH USA

**Keywords:** Hypoalbuminemia, COVID-19, SARS-CoV-2, Severe disease

The coronavirus disease 2019 (COVID-19) pandemic necessitates identifying laboratory markers to assist the clinicians in early recognition of severe disease [1]. Given the unclear association of hypoalbuminemia and severe COVID-19, we conducted a systematic review and meta-analysis to answer this.

An extensive literature search of PubMed/MEDLINE, Embase, Cochrane, and Web of Science was conducted through April 3, 2020, using search strategy created by an experienced librarian (W.L.S). Two independent reviewers (M.A. and R.F.) performed screening and data extraction of articles. Articles were selected if they reported data on COVID-19 patients with respect to hypoalbuminemia. Discrepancy in screening/data collection was resolved through mutual discussion. Random-effects meta-analysis was conducted, and odds ratio (OR) and mean difference (MD) for proportional and continuous variables were computed, respectively. For each outcome, forest plot, 95% confidence interval (CI), *p* value (< 0.05 considered statistically significant), and *I*^2^ statistic (> 50% considered as substantial heterogeneity) was generated using Open Meta Analyst (CEBM, Oxford, UK).

Severe COVID-19 was defined as respiratory distress (with either rate ≥ 30/min, oxygen saturation ≤ 93% at rest, and/or PaO2/FiO2 ≤ 300 mmHg), ICU admission, and/or death [1]. Hypoalbuminemia was reported based on reference laboratory parameters for each study.

A total of 11 studies (with 910 patients, mean age 47.6 ± 8.2 years and 47.0% females) were included (Table 1). The weighted mean serum albumin on admission was 3.50 g/dL (CI 3.26–3.74 g/dL) and 4.05 g/dL (CI 3.82–4.27 g/dL) in severe and non-severe COVID-19 group, respectively. This was statistically significant (MD:− 0.56 g/dL, CI -0.69 to -0.42 g/dL, *p* < 0.001, *I*^2^ = 91.2%)(Fig. 1a). Leave-one-out meta-analysis was consistent with point estimate (MD) ranging from -0.61 to -0.51 g/dL (Fig. 1b). The results were consistent on subgroup analysis of 8 studies that defined severe COVID-19 based on respiratory distress definition (MD -0.58 g/dL, 95% CI -0.78 to -0.37 g/dL, *p* < 0.001, *I*^2^ = 87.9%). Four studies assessed the hypoalbuminemia status and severe COVID-19 and increased risk was demonstrated (OR 12.6, 95% CI 7.5–21.1, *p* < 0.001, *I*^2^ = 0%) (Fig. 1c).

**Table 1:** Study characteristic and demographics of included patients (*n* no. of patients, *NR* not reported, *SD* standard deviation)

Study, year	Country	Language	Hospital	Study period	Total patients	Mean/median age	Female gender,*n* (%)	Severe patients^#^, *n* (%)	Serum albumin level, mean (SD) g/dL	
									Severe	Non-severe
Huang, 2020 [2]	China	English	Jinyintan Hospital	Dec 16 to Jan 2	41	49	11 (26.8%)	13 (31.7%)	2.83 (0.24)	3.4 (0.27)
Chen (1), 2020 [3]	China	English	Tongji Hospital	Jan 13 to Feb 28	274	NR	NR	113 (41.2%)	3.03 (0.06)	3.65 (0.26)
Liu (1), 2020 [4]	China	English	Shenzhen Third People’s Hospital	Jan 11 to Jan 21	12	58.9	4 (33.3%)	6 (50.0%)	3.77 (0.25)	4.43 (0.34)
Chen (2), 2020 [5]	China	English	Tongji Hospital	Dec to Jan 27	21	56	4 (19.0%)	11 (52.4%)	3.02 (0.24)	3.73 (0.22)
Mo, 2020 [6]	China	English	Zhongnan Hospital	Jan 1 to Feb 5	155	54	69 (44.5%)	92 (59.4%)	3.6 (0.31)	3.9 (0.27)
Wan, 2020 [7]	China	English	Chongqing University Three Gorges Hospital	Jan 23 to Feb 8	135	47	63 (46.7%)	40 (29.6%)	3.59 (0.26)	4.52 (0.27)
Liu (2), 2020 [8]	China	Chinese	Multicenter	Jan 23 to Feb 8	32	38.5	12 (37.5%)	4 (12.5%)	3.55 (0.44)	4.05 (0.34)
Liu (3), 2020 [9]	China	Chinese	Jianghan University Affiliated Hospital	Jan 10 to Jan 31	30	35	20 (66.7%)	4 (13.3%)	3.5 (0.21)	4.2 (0.28)
Liu (4), 2020 [10]	China	English	Multicenter	Dec 30 to Jan 15	78	38	39 (50.0%)	11 (14.1%)	3.66 (0.43)	4.13 (0.33)
Zhang, 2020 [11]	China	English	Zhongnan Hospital	Jan 18 to Feb 22	115	49.52	66 (57.4%)	31 (30.0%)	3.44 (0.31)	4.04 (0.28)
Zhou, 2020 [12]	China	English	Ninth Hospital of Nanchang	Jan 28 to Feb 6	17	41.7	11 (64.7%)	5 (29.4%)	4.6 (0.28)	4.49 (0.27)

^#^Respiratory distress (rate ≥ 30/min, oxygen saturation ≤ 93% at rest and/or PaO2/FiO2 ≤ 300 mmHg), *ICU* admission and/or death

**Fig. 1:**
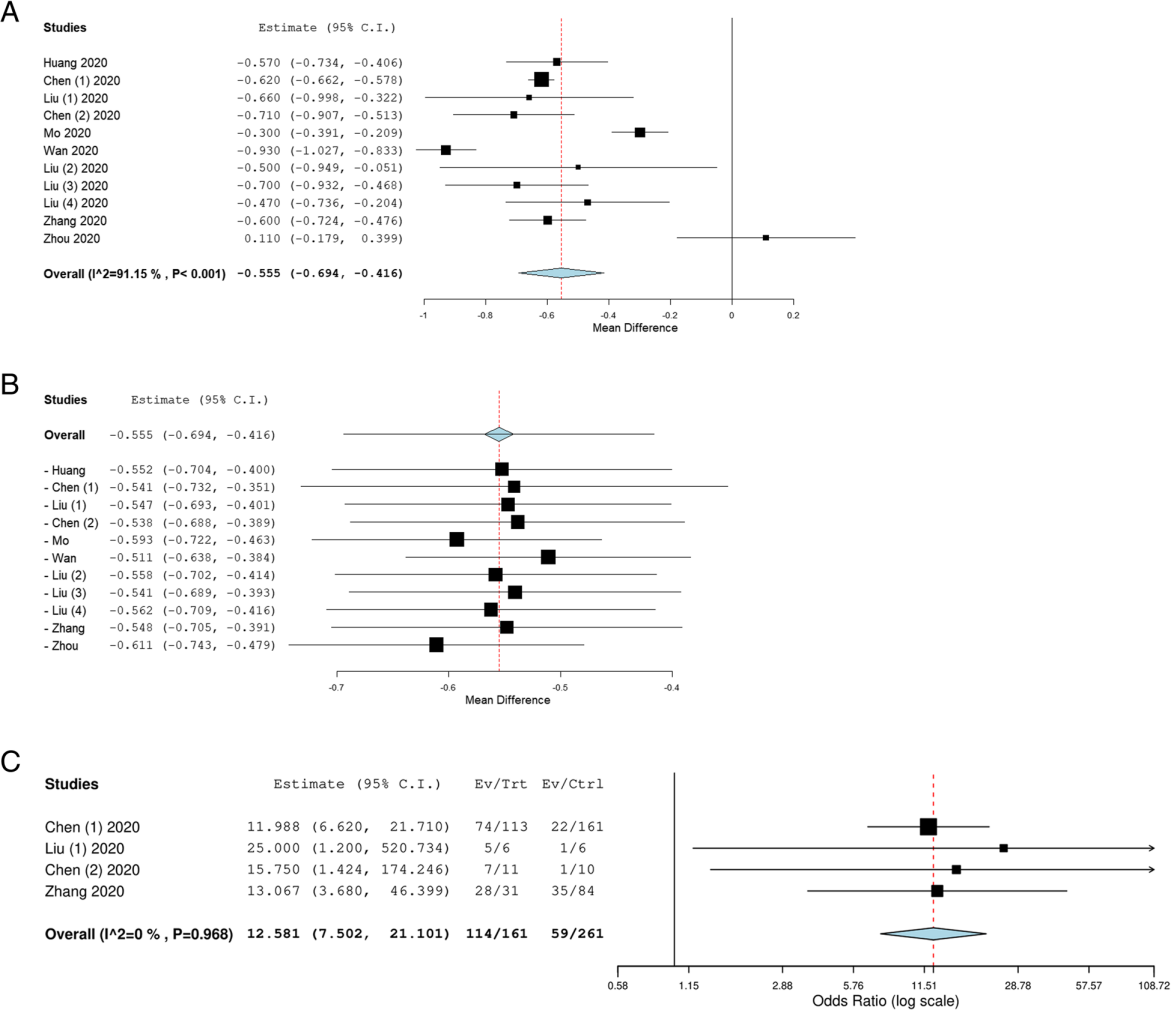
Forest plot demonstrating **a** meta-analysis comparing mean serum albumin, **b** leave-one-out meta-analysis comparing mean serum albumin, and **c** meta-analysis comparing hypoalbuminemia status for patients in severe vs non-severe group (C.I. confidence interval)

Hypoalbuminemia status has been associated with critically ill patients and mortality across numerous clinical settings [13]. The pathophysiology behind hypoalbuminemia in disease state (such as pancreatitis, infection, trauma, burn, and organ dysfunction) is thought to be secondary to increased capillary permeability, decreased protein synthesis, decreased half-life of serum albumin, decreased serum albumin total mass, increased volume of distribution, and increase expression of vascular endothelial growth factor [14]. The hallmark of severe COVID-19 includes the cytokine storm and an interplay of some of the aforementioned mechanisms [1].

Our study had some limitations. There was lack of reporting on temporal association of hypoalbuminemia and severe COVID-19. The serum albumin level was noted on admission; however, it is difficult to make conclusive evidence whether severe COVID-19 caused hypoalbuminemia or vice versa. We were also not able to address if hypoalbuminemia should be corrected or not in the current study and needs further evaluation in future studies. The strength of our study is the reporting of large cohort of patients with consistent results across subgroup and sensitivity analysis.

We demonstrate the association of hypoalbuminemia and severe COVID-19. A low albumin level can potentially lead to early recognition of severe disease and assist clinicians in making informed decision for their patients.

## Data Availability

The datasets used and/or analyzed during the current study are available from the corresponding author on reasonable request.

## Abbreviations

CI: Confidence interval
COVID-19: Coronavirus disease 2019
ICU: Intensive care unit
MD: Mean difference
*n*: No. of patients
OR: odds ratio
SD: standard deviation
